# Elucidating the mechanism of Hongjinshen decoction in the treatment of pulmonary fibrosis based on network pharmacology and molecular docking

**DOI:** 10.1097/MD.0000000000032323

**Published:** 2022-12-23

**Authors:** Haixu Chen, Yu Lin, Lianlin Zeng, Shiwei Liu

**Affiliations:** a Department of Basic Medicine, Sichuan Vocational College of Health and Rehabilitation, Zigong, Sichuan, China; b Department of Rehabilitation Medicine, Zigong No.4 People’s Hospital, Sichuan, China; c Department of Human Anatomy and Histoembryology, School of Basic Medical Sciences, Southwest Medical University, Luzhou, China; d Department of Clinical and Medical Technology, Sichuan Vocational College of Health and Rehabilitation, Zigong, Sichuan, China.

**Keywords:** and nourish yin, boost qi, Compound Hongginshen Decoction, network pharmacology, pulmonary fibrosis

## Abstract

To explore the mechanism of compound Hongginshen decoction in improving pulmonary fibrosis based on network pharmacology.

**Methods:**

The active components and targets of ginseng and Salvia miltiorrhiza were screened from the Traditional Chinese Medicine Systems Pharmacology Database and Analysis Platform (TCMSP) database. The chemical components of Rhodiola, Ophiopogon japonicus, and Dendrobium were screened using the Traditional Chinese Medicine Integrated Database (TCMID), and the target compounds were predicted by the Swisstargets method. The related target genes of pulmonary fiber (PF) were screened by the Genecards database and the National Center of Biotechnology Information (NCBI) database. The protein–protein interaction network was drawn using the string database and Cytoscape software, and the network topology was analyzed. Then, using R3.6.3 software, biological processes, molecular function, cell component enrichment, and Kyoto Encyclopedia of Genes and Genomes (KEGG) pathway enrichment were carried out on the common targets of drugs and diseases. The network diagram of the “traditional Chinese medicine composition disease target” of Compound Hongginshen Decoction was constructed and analyzed with the software of Cytoscape 3.6.1.

**Results:**

We identified 159 active components and 2820 targets in Compound Hongginshen Decoction, and 2680 targets in pulmonary fibrosis. A total of 343 common targets were obtained by the intersection of drug targets and disease targets. protein–protein interaction protein interaction network analysis showed that PIK3CA, PIK3R1, MAPK1, SRC, AKT1, and so on may be the core targets of the compound Hongjingshen recipe in the treatment of pulmonary fibrosis. Gene Ontology (GO) enrichment analysis identified 3463 items, and KEGG pathway enrichment analysis identified 181 related signaling pathways, including the PI3K-Akt signaling pathway, HCMV pathway, Hb pathway, PGs pathway, and KSHV signaling pathway.

**Conclusion:**

Compound Hongginshen Decoction has the characteristics of a multichannel and multitargeted effect in the treatment of pulmonary fibrosis. Radix Ophiopogonis and Dendrobium officinale play a key role in the treatment of pulmonary fibrosis. The whole compound prescription may play a therapeutic role by affecting cell metabolism, being anti-inflammatory, regulating the immune system, promoting angiogenesis, and improving anaerobic metabolism.

## 1. Introduction

Pulmonary fibrosis (PF) is an interstitial lung disease caused with various pathogenic factors both inside and outside the lung and is characterized by the infiltration of inflammatory cells such as macrophages and lymphocytes in the interstitium, proliferation of fibroblasts, and deposition of fibrous connective tissue in the interstitium.^[1]^ Abnormal proliferation of lung fibroblasts and excessive deposition of collagen-based extracellular matrix are key pathogenic aspects of PE. However, the complete molecular mechanisms involved are still unclear.^[2]^ Pulmonary fibrosis is an end-stage disease of the lung and is characterized by coughing, progressive dyspnea, restricted mobility, and other symptoms, and is often treated clinically with a combination of glucocorticoids and anti-infectives, antipulmonary fibrosis drugs, and immunosuppressive drugs, or in more severe cases, lung transplantation. Unfortunately, these treatments suffer from long treatment cycles and significant side effects. Chinese medicine focuses on the unity of the whole and the local, the connection and the development, and integrates the interrelationship between disease and medicine, which not only has a rich practical basis but also has the advantages of less adverse effects and milder drug action.

Pulmonary fibrosis does not have a specific name in Chinese medicine, but most practitioners classify it as “panting syndrome,” “phlegm rheum,” “cough,” “lung atrophy,” “lung distention,” “lung bi”, and “shortness of breath” based on its clinical manifestations. Some scholars believe that it belongs to the category of Chinese medicine’s luo disease. Other scholars believe that the early stage of PF is mainly due to the actual evidence of lung qi block and can be classified as “lung bi,” while the late stage of the disease is mainly due to the impairment of lung, spleen, and kidney functions, and further development leads to weakening of the lung lobes and loss of their ascending and descending functions, which can eventually be classified as “lung atrophy.” Based on the clinical manifestations of PF and the differences in traditional Chinese medicine (TCM) treatment at different stages, we believe that it belongs to both the “paralysis” and “atrophy” categories, and that it is a combination of both deficiency and excess. The Synopsis of the Golden Chamber was the first to record the name “lung atrophy,” with the comment that if a patient presents symptoms such as “rapid pulse in the radial pulse,” “cough,” and “Coughing up mucous sputum,” then the disease is “lung atrophy.” Liu Guanru et al proposed that “lung atrophy” is mainly caused by externally contracted pathogenic qi, internal damage caused by the 7 emotions, alcoholism, qi deficiency, and sexuality, and leads to the accumulation of pathological products such as phlegm, static blood, and toxin in the body. In the early stage of the disease, dysfunction of lung qi is the main cause, and in the later stage, lung–kidney vacuity is the main cause, which can be summarized as primary asthenia-secondary asthenia syndrome, and based on lung–kidney vacuity, phlegm, and blood stasis throughout the course of the disease. Some TCM scholars have summarized the etiology, disease mechanism, and treatment of “lung atrophy” at different stages: in the early stage, it can be classified as “lung bi,” in the middle stage, it is “lung bi” and “lung atrophy,” and “lung atrophy” in the late stage. The treatment strategy can be formulated according to the etiology and disease mechanism at different stages. The disease mechanism is qi (yin) deficiency and blood stasis, and the treatment is often accompanied by the use of supplements to the lung to boost qi, nourish yin to moisten the lung, invigorate blood, and dissolve stasis. In a TCM 4-examination data set containing 131 patients with idiopathic pulmonary fibrosis, we found by stratified clustering of indicator variables that 35.1% were lung–kidney qi deficiency, 16.8% were deficiency of both qi and yin, 19.1% were heart qi deficiency, 17.6% were qi stagnation and blood stasis, and 11.4% were lung-spleen qi deficiency. In conclusion, Qi deficiency and Yin deficiency are the main components of the TCM etiology and pathogenesis of pulmonary fibrosis. Some TCM scholars believe that exploring nourishing Yin and benefiting Qi class of herbs for pulmonary fibrosis compared to conventional Western medicine treatment, Chinese medicine nourishes Yin and benefits Qi method is more effective for idiopathic pulmonary fibrosis, which mentions several herbs such as Ophiopogon japonicus, Salvia miltiorrhiza, and Schisandra chinensis. Therefore, in summary, the treatment of pulmonary fibrosis is mainly based on the treatment of Qi, Blood, and Yin.

The Compound Hongginshen Decoction is derived from Shengmai Yin plus and minus, which consists of Rhodiola, Ginseng, Ophiopogon japonicus, Dendrobium, and Salvia miltiorrhiza, with Rhodiola and Ginseng as the chief medicinals, Ophiopogon japonicus and Dendrobium as the deputy medicinals, and Salvia miltiorrhiza as the assistant medicinals. Rhodiola belongs to the heart and lung meridians. It has the effects of boosting qi and invigorating blood, opening the veins, and being anti-asthmatic. Ginseng belongs to the heart, lung, spleen, and kidney meridians, and has the function of tonifying the spleen and lung, enriching yin, nourishing the blood and providing powerful supplementation of original qi. The combination of Ophiopogon japonicus and Dendrobium can strengthen the power of nourishing Yin and fluid, nourishing Yin, and clearing heat, whereas the combination with Salvia miltiorrhiza can invigorate blood and dissolve stasis, as well as improve the function of Rhodiola and Ginseng to move qi and dredge collaterals. The Compound Hongginshen Decoction embodies the 4-in-one treatment concept of dissolving stasis, moving qi, supplementing qi, and promoting fluid production. Compared with the ancient formula of Shengmai Yin, both formulas have the effects of moving qi, boosting qi, and nourishing Yin, but the new formula of Compound Hongginshen Decoction enhances the functions of moving qi and nourishing Yin and increases the effect of invigorating blood and dissolving stasis. However, at this stage, it is not clear what the chemical composition of the Compound Hongginshen Decoction is or how it exerts its mechanism of action in the treatment of pulmonary fibrosis. Network pharmacology is based on a multi-level network of multiple genes, pathways, and pathways and is used to study traditional Chinese medicine from a holistic perspective, which has unique advantages. Therefore, in this study, a network pharmacology approach was used to investigate the main active components and mechanisms of action of the Compound Hongginshen Decoction to improve pulmonary fibrosis.

## 2. Materials and Methods

### 2.1. Screening of drug-related active compounds and potential targets

The Traditional Chinese Medicine Systems Pharmacology Database and Analysis Platform (TCMSP) database (https://tcmspw.com/tcmsp.php) was searched for ginseng, salvia components and targets, and the screening conditions for the components were set to oral bioavailability ≥ 30% and drug-like properties ≥ 0. 18%. The active ingredients of Rhodiola, Ophiopogon japonicus, and Dendrobium were obtained from the Traditional Chinese Medicine Integrated Database (TCMID) database (http://119.3.41.228:8000/tcmid/herb/6636/) because they were not available in the TCMSP database, and then the various active ingredients of the 3 herbs were obtained from the Swisstargets database (http://www.swisstargetprediction.ch/) to obtain the corresponding ADME parameters, and the ingredients with better oral utilization and drug-like properties were included, and finally the potential targets of individual active ingredients were predicted. In addition, we also extracted the pharmacologically active chemical components in conjunction with the 2020 edition of the Chinese Pharmacopoeia and literature reports.

### 2.2. Prediction of PF targets

The keyword “pulmonary fibrosis” was used in GeneCards (https://www.genecards.org/), National Center of Biotechnology Information (NCBI) gene database (https://www.ncbi.nlm.nih.gov/) for human gene searches. The GeneCards data were screened for a median relevance score, and targets above the median were included to obtain more relevant disease targets.

### 2.3. Acquisition of drug–disease common targets

The screened drug targets and disease targets are entered into Venn diagram creation software (Venny 2.1) to form an intersection analysis diagram of drug targets and disease targets.

### 2.4. Protein–protein interaction network construction and screening of core genes

The drug–disease common target information was entered into the String database (https://string-db.org/cgi/input.pl), and the biological species was set to “Homo sapiens,” the minimum interaction critical value was set to “highest confidence” (> 0.9), and the rest settings were set as default to obtain the protein–protein interaction (PPI) network. The PPI network was imported into Cystoscap 3.6.1, and topology analysis was performed by the NetworkAnalyzer tool. Then the 4 parameters degree, betweenness centrality, average shortest path length, and closeness centrality were used as reference standards, and the genes with scores greater than the average score were selected as key targets by sorting the degree and using R 3.6.3. The visualization was performed.

### 2.5. GO function and KEGG pathway enrichment analysis

The drug–disease common targets were enriched for biological process (BP), molecular function (MF), and cell component (CC) of GO, and items with a corrected *P*-value ≤ 0.05 were screened by referring to the String database. Then the clusterProfiler, enrichplot, and ggplot2 packages of R 3.6.3 software were applied for histogram and bubble plotting. The drug–disease common targets were subjected to Kyoto Encyclopedia of Genes and Genomes (KEGG) enrichment analysis while the String database was referenced and items with corrected P-values ≤ 0.05 were screened. Histograms and bubble plots were then performed using the clusterProfiler package of R 3.6.3.

### 2.6. Construction of drug–targets disease network

In order to better understand the complex interactions between herbal medicines, active ingredients, diseases, and corresponding targets, an active ingredient–disease target network diagram was constructed based on the incorporated corresponding herbal medicines, active ingredients, diseases, and targets and imported into cytoscape 3.6.1 for network mapping. Finally, the key ingredients were ranked by degree to obtain the key active ingredients.

### 2.7. Ethical statement

This study does not include studies of human subjects, human data, or human tissues or animals. All data is obtained from the database, so ethical certification is not required.

## 3. Result

### 3.1. Screening and collection of active ingredients and potential targets of compound Red Ginseng Formula

The active ingredients of Ginseng and Salvia miltiorrhiza were obtained by screening through the TCMSP database (with oral bioavailability ≥ 30% and drug-like properties ≥ 0.18 as the screening conditions), together with NCBI, the Chinese Pharmacopoeia, and related literature reports. The active ingredients of Rhodiola, Dendrobium, and Ophiopogon japonicus were screened by the TCMID database. Then the Swisstargets database was used to predict the potential targets of various active ingredients. Finally, as shown in Table 1, 159 active ingredients were obtained (20 for Rhodiola, 22 for Ginseng, 23 for Dendrobium, 30 for Ophiopogon japonicus, and 64 for Salvia miltiorrhiza), and there was an overlap of active ingredients contained between different herbal medicines, and the duplicate values were removed.

**Table 1:** Ingredients (species) and targets (per) of Compound Hongginshen Decoction.

Chinese medicinal	Ingredients/species	Targets/per
Rhodiola	20	599
Ginseng	22	256
Salvia miltiorrhiza	64	935
Dendrobium	23	541
Ophiopogon japonicus	30	492

### 3.2. Identification of PF targets

Using “pulmonary fibrosis” as the keyword, 2574 genes were retrieved from the GeneCards database and 425 genes from the NCBI database. Then, after combining the genes obtained from these 2 databases and removing duplicates, 2680 pulmonary fibrosis-related genes were obtained.

### 3.3. Identification of common drug–disease targets

**Figure 1.**
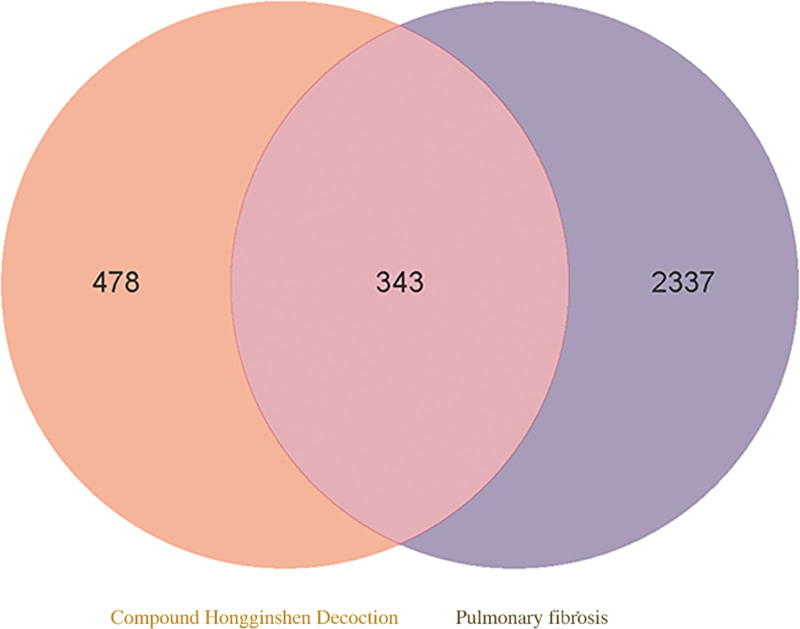
Venn diagram of drugs and disease. Orange is the herbal compound target; lavender is the pulmonary fibrosis target; middle pink is the duplicate item.

The screened potential drug targets (821 for herbal compounds) and disease targets (2680 for PF) were entered into Venny 2.1, the software for creating Venn diagrams, resulting in 343 common target targets, 478 drug-unique targets and 2337 disease-unique targets (Fig. 1).

### 3.4. PPI network construction and core genes selection

**Figure 2.**
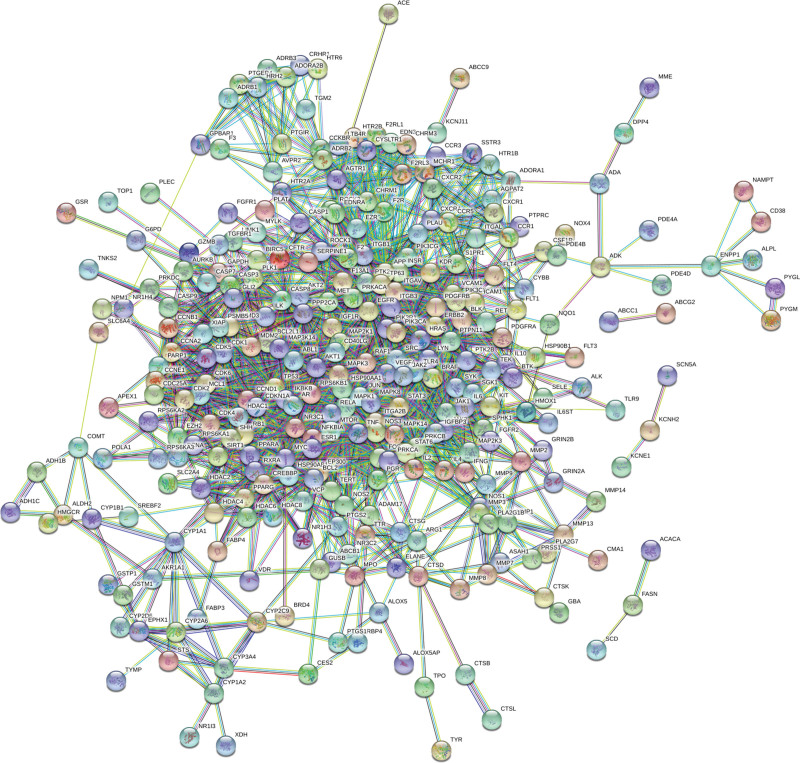
PPI network diagram of common drug–disease targets. PPI = protein–protein interaction.

**Figure 3.**
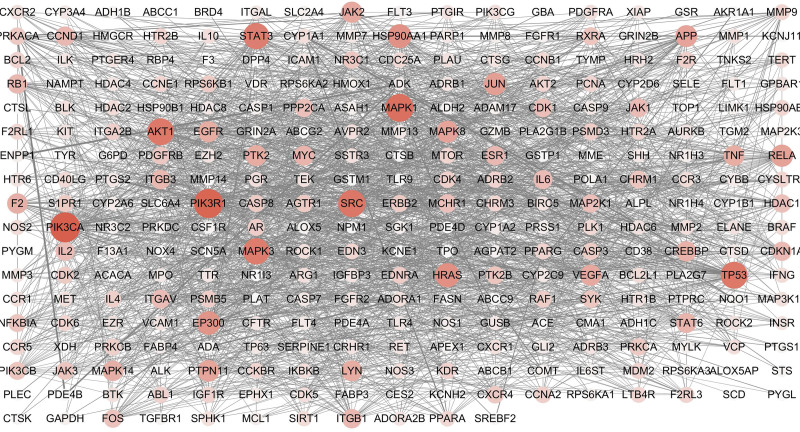
PPI network visualization analyses. The nodes represent the targets, and the size and color shades show their degree in the network. PPI = protein–protein interaction.

**Figure 4.**
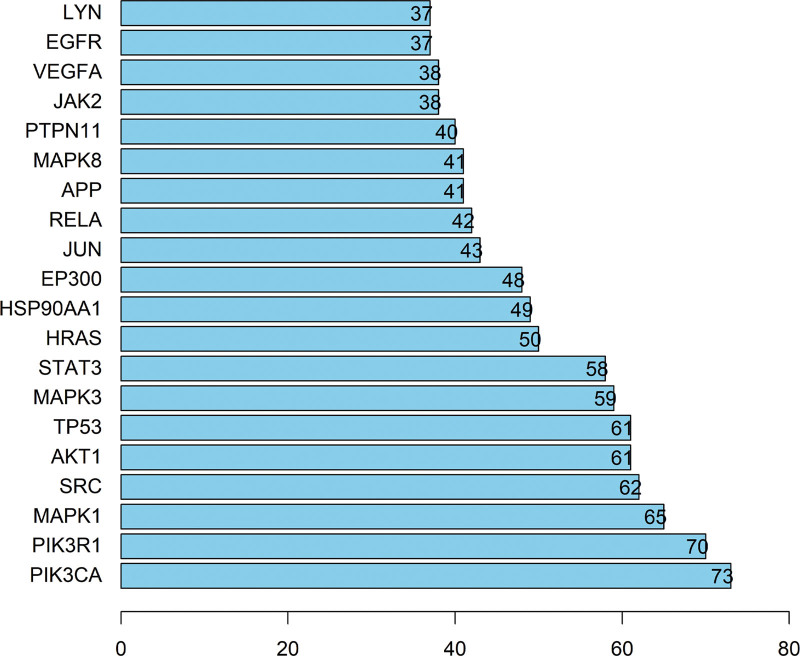
Screening of the top 20 genes based on the PPI topology analysis for degree. The horizontal coordinate represents the value of degree and the vertical coordinate represents the specific target name. PPI = protein–protein interaction.

The drug-disease common target was entered into the String database, then the biological species was set to Homo sapiens, the minimum interaction threshold was set to the highest confidence level (≥0.9), and the rest of the settings were set as default. We obtained the PPI network, which contained 343 nodes, 2070 edges, and a mean degree value of 12.1 (Figs. 2 and 3). The PPI network was imported into Cystoscap 3.6.0, and topology analysis was performed by the NetworkAnalyzer tool. We used the 4 parameters degree, betweenness centrality, average shortest path length, and closeness centrality as reference criteria, and selected genes with scores greater than the average score as key targets by degree ranking. Finally, a total of 110 key targets were selected, and the targets were ranked from highest to lowest by degree, such as PIK3CA, PIK3R1, MAPK1, SRC, AKT1, and TP53. After network topology analysis, the key targets were PIK3CA, PIK3R1, MAPK1, SRC, AKT1, etc., which play important roles in cell growth and apoptosis, in order of core degree from highest to lowest. The top 20 targets were visualized using R 3.6.3, and the horizontal coordinates in the graph are the degree values of each target (Fig. 4).

### 3.5. GO and KEGG enrichment analysis of drug–disease common targets

**Figure 5.**
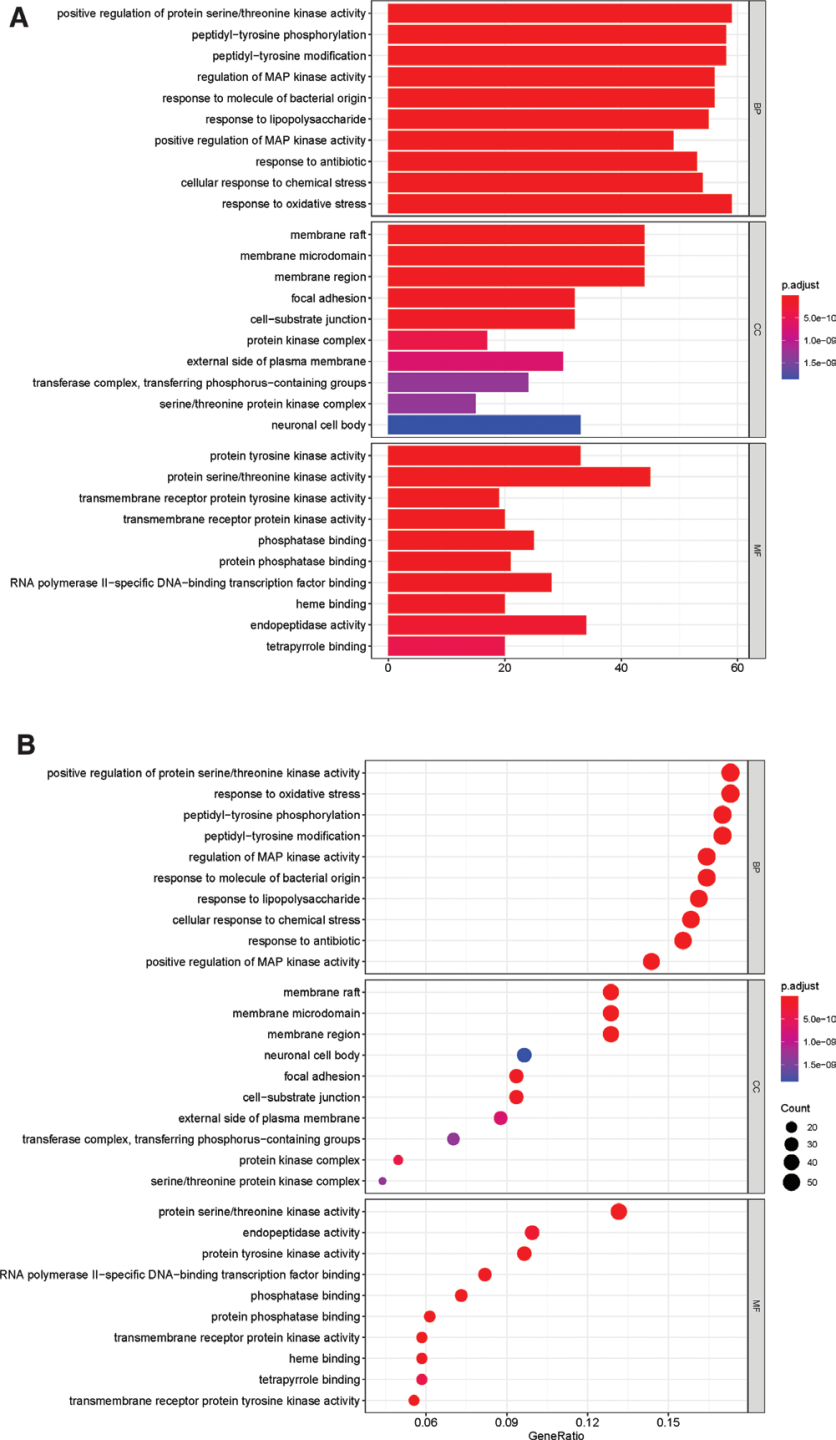
GO functional enrichment analyses of common genes. Significant enrichment of common genes in MF (molecular function) category (a), CC (cellular component) category (b), and BP (biological process) category (c) (the top 10 GO terms for each category are listed).

**Figure 6.**
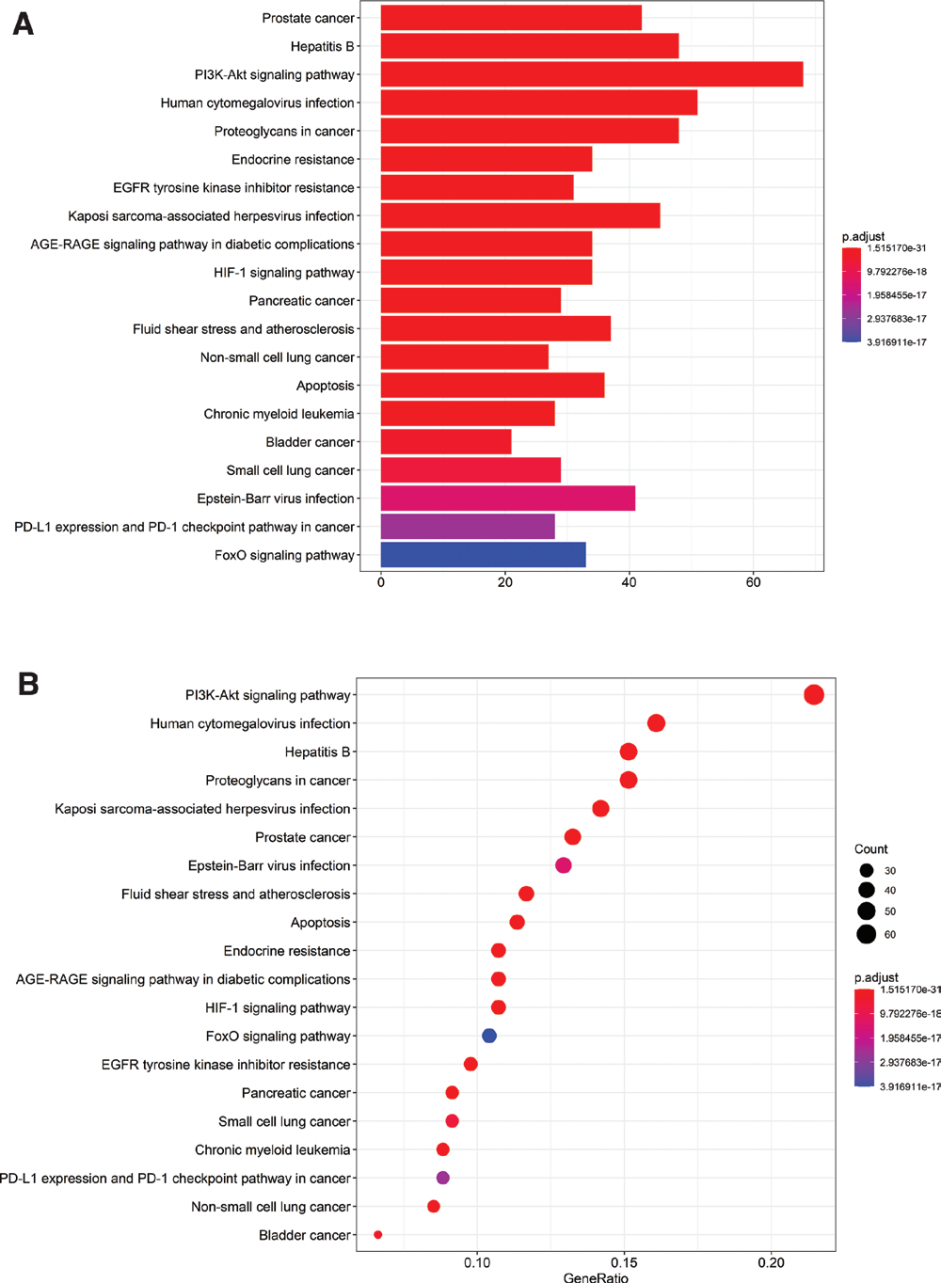
KEGG pathway enrichment analysis of common genes. Red indicates small P-values, blue indicates large P-values; the size of the bars represents the degree of enrichment.

Enrichment of GO for BP, MF, and CC for drug-disease common targets, and screening of items with corrected *P*-value ≤ 0.05 by using the String database. We enriched a total of 3094 BPs of interest, 243 MFs of relevance, and 126 cellular compositions. Then the top 10 significant BPs, MFs, and CCs were selected for each, and then the clusterProfiler, enrichplot, and ggplot2 packages in R 3.6.3 software were used in order to plot bar and bubble plots (Figs. 5A and B). According to GO enrichment importance, the top 30 relevant pathways and their involved genes were listed (Table 2). After GO analysis, it was concluded that the common disease–drug targets were mainly enriched in positive regulation of protein serine/threonine kinase activity, peptidyl-tyrosine phosphorylation, peptidyl-tyrosine modification, regulation of MAP kinase activity, response to molecules of bacterial origin, etc. The drug–disease common targets were subjected to KEGG enrichment analysis, selecting items with corrected p-values ≤ 0.05 and enriching a total of 181 signaling pathways (Table 3). The top 20 highly significant signaling pathways were selected, and the clusterProfiler package in R 3.6.3 was used to plot bar and bubble plots (Figs. 6A and B). The KEGG enrichment analysis revealed that the drug-disease common targets are mainly enriched in the following signaling pathways: PI3K-Art signaling pathway, HCMV signaling pathway, HB signaling pathway, PGS pathway, and KSHV pathway. These pathways play an important role in cell growth, proliferation, survival, inhibition, migration, and metabolism.

**Table 2:** Gene Ontology-related genes.

ID	Description	geneID
GO:0071902	positive regulation of protein serine/threonine kinase activity	VEGFA/TNF/AKT1/ERBB2/CXCR4/TLR4/IFNG/CCND1/JAK2/MAPK1/SIRT1/MIF/EZH2/TLR9/PTPN11/F2R/PDGFRB/NOX4/PIK3CG/PPP2CA/HSP90AB1/ADORA2B/TGFBR1/ELANE/ADRB2/SRC/CD40LG/KIT/EGFR/HRAS/FGFR1/PTPRC/MAP2K1/RAF1/PDE5A/MAPK14/RET/INSR/ADORA1/HTR2B/FLT1/MAPK3/P2RX7/SYK/HTR2A/ILK/FLT3/PLA2G1B/CDK1/CSF1R/ALK/PTK2B/CCNB1/PIK3CB/MAP2K3/EDN3/ERN1/MAP3K14/ADAM17
GO:0018108	peptidyl-tyrosine phosphorylation	TP53/VEGFA/IL6/TNF/STAT3/ERBB2/ACE/IFNG/JAK2/IL4/MIF/ITGB3/PTPN11/PDGFRB/NOX4/PPP2CA/IL6ST/SRC/PDGFRA/KIT/EGFR/FGFR1/PTPRC/DDR1/TEK/ENPP2/ICAM1/MAP2K1/NEK1/IL2/FGFR2/HDAC2/MET/MTOR/KDR/IGF1R/RET/INSR/ADORA1/FLT1/MAPK3/JAK1/JAK3/PTK2/BTK/SYK/HTR2A/ABL1/LYN/FLT4/FLT3/CSF1R/ALK/PTK2B/MAP2K3/BLK/APP/ADAM17
GO:0018212	peptidyl-tyrosine modification	TP53/VEGFA/IL6/TNF/STAT3/ERBB2/ACE/IFNG/JAK2/IL4/MIF/ITGB3/PTPN11/PDGFRB/NOX4/PPP2CA/IL6ST/SRC/PDGFRA/KIT/EGFR/FGFR1/PTPRC/DDR1/TEK/ENPP2/ICAM1/MAP2K1/NEK1/IL2/FGFR2/HDAC2/MET/MTOR/KDR/IGF1R/RET/INSR/ADORA1/FLT1/MAPK3/JAK1/JAK3/PTK2/BTK/SYK/HTR2A/ABL1/LYN/FLT4/FLT3/CSF1R/ALK/PTK2B/MAP2K3/BLK/APP/ADAM17
GO:0043405	regulation of MAP kinase activity	VEGFA/TNF/ERBB2/CXCR4/TLR4/JAK2/MAPK1/MIF/EZH2/TLR9/PTPN11/F2R/PDGFRB/NOX4/PIK3CG/PPP2CA/ADORA2B/TGFBR1/ELANE/SRC/CD40LG/KIT/EGFR/HRAS/FGFR1/PTPN22/PTPRC/GBA/MAP2K1/RAF1/PDE5A/MAPK14/IGF1R/RET/GSTP1/INSR/ADORA1/HMGCR/HTR2B/FLT1/MAPK3/P2RX7/SYK/HTR2A/LYN/ILK/FLT3/PLA2G1B/CDK1/ALK/PTK2B/PIK3CB/MAP2K3/EDN3/ERN1/MAP3K14
GO:0002237	response to molecule of bacterial origin	IL6/TNF/AKT1/PTGS2/TLR4/JAK2/MAPK1/SERPINE1/MIF/JUN/TLR9/MAPK8/F2R/VCAM1/FOS/IL10/ELANE/SRC/MPO/NR1H4/PTPN22/ICAM1/NOS3/NOS2/FGFR2/CTSG/HDAC2/CASP8/CCR5/SELE/MAPK14/GSTP1/CASP3/CYP1A1/ALPL/COMT/PDE4D/MAPK3/CDK4/NFKBIA/RELA/P2RX7/CASP1/ARG1/NR1H3/PRKCA/ABL1/PTGER4/NOS1/LYN/CASP9/PDE4B/RPS6KA3/CYP1A2/PTGIR/ADAM17
GO:0032496	response to lipopolysaccharide	IL6/TNF/AKT1/PTGS2/TLR4/JAK2/MAPK1/SERPINE1/MIF/JUN/MAPK8/F2R/VCAM1/FOS/IL10/ELANE/SRC/MPO/NR1H4/PTPN22/ICAM1/NOS3/NOS2/FGFR2/CTSG/HDAC2/CASP8/CCR5/SELE/MAPK14/GSTP1/CASP3/CYP1A1/ALPL/COMT/PDE4D/MAPK3/CDK4/NFKBIA/RELA/P2RX7/CASP1/ARG1/NR1H3/PRKCA/ABL1/PTGER4/NOS1/LYN/CASP9/PDE4B/RPS6KA3/CYP1A2/PTGIR/ADAM17
GO:0043406	positive regulation of MAP kinase activity	VEGFA/TNF/ERBB2/CXCR4/TLR4/JAK2/MAPK1/MIF/EZH2/TLR9/PTPN11/F2R/PDGFRB/NOX4/PIK3CG/ADORA2B/TGFBR1/ELANE/SRC/CD40LG/KIT/EGFR/HRAS/FGFR1/PTPRC/MAP2K1/RAF1/PDE5A/MAPK14/RET/INSR/ADORA1/HTR2B/FLT1/MAPK3/P2RX7/SYK/HTR2A/ILK/FLT3/PLA2G1B/CDK1/ALK/PTK2B/PIK3CB/MAP2K3/EDN3/ERN1/MAP3K14
GO:0046677	response to antibiotic	TP53/IL6/STAT3/CRHR1/CCND1/JAK2/SIRT1/HMOX1/EZH2/JUN/HSP90AA1/VCAM1/PDGFRB/CFTR/IL10/SRC/ADA/ICAM1/STAT6/IL2/FGFR2/HDAC2/MET/CASP8/GSTP1/CASP3/APEX1/CYP1A1/ALPL/G6PD/HMGCR/MDM2/RBP4/BCL2/BCL2L1/RELA/JAK1/ARG1/ABL1/CYBB/GRIN2B/CASP9/PCNA/NQO1/CDK1/HDAC6/SLC2A4/CYP1B1/GRIN2A/HTR3A/PTK2B/HTR1B/SPHK1
GO:0062197	cellular response to chemical stress	TP53/IL6/TNF/MMP9/AKT1/PTGS2/TLR4/PPARG/JAK2/MMP2/MAPK1/SIRT1/HMOX1/EZH2/JUN/MMP3/MAPK8/NOX4/FOS/IL10/SRC/MPO/PDGFRA/EGFR/STAT6/NOS3/HDAC2/MET/GSTP1/CASP3/APEX1/MYLK/G6PD/PARP1/MDM2/GSR/BCL2/MAPK3/RELA/MCL1/BTK/ARG1/ABL1/CYBB/EIF2AK3/PCNA/NQO1/CDK1/HDAC6/SLC2A4/CYP1B1/CDK2/CCNA2/SPHK1
GO:0006979	response to oxidative stress	TP53/IL6/TNF/MMP9/AKT1/PTGS2/TLR4/JAK2/MMP2/MAPK1/SIRT1/HMOX1/EZH2/JUN/MMP3/MAPK8/PDGFRB/NOX4/FOS/IL10/SRC/MPO/PDGFRA/EGFR/ADA/STAT6/NOS3/HDAC2/MET/GSTP1/CASP3/APEX1/G6PD/PARP1/MDM2/PSMB5/GSR/TPO/BCL2/MAPK3/PTGS1/RELA/MMP14/MCL1/BTK/ARG1/ABL1/CYBB/PCNA/NQO1/CDK1/HDAC6/CYP1B1/CDK2/PTK2B/CCNA2/APP/SPHK1/CD38
GO:0035690	cellular response to drug	TP53/IL6/TNF/CXCR4/PTGS2/MAPK1/SIRT1/HMOX1/EZH2/MMP3/ROCK2/HSP90AB1/CFTR/IL10/SRC/EGFR/PTPN22/ICAM1/STAT6/CHRM3/NOS2/HDAC2/PDE4A/MET/KDR/APEX1/NR3C1/MYC/MDM2/HTR2B/TRPV1/MAPK3/CDK4/RELA/P2RX7/ARG1/CHRM1/HRH2/HTR2A/ABL1/KCNH2/CYBB/CASP9/PDE4B/PCNA/NQO1/CDK1/HDAC6/CYP1B1/ALK/KCNJ11/HTR3A/CDK2/HTR1B/CCNA2/HTR6/SPHK1/HSP90B1
GO:0018105	peptidyl-serine phosphorylation	VEGFA/IL6/TNF/AKT1/PTGS2/BRAF/IFNG/MAPK1/MIF/HSP90AA1/MAPK8/PRKDC/TOP1/AKT2/ROCK2/HSP90AB1/TGFBR1/SRC/EGFR/PIK3CA/RAF1/MTOR/MAPK14/RET/SGK1/PDE4D/BCL2/MAPK3/PRKACA/IKBKB/RPS6KB1/PLK1/SYK/PRKCA/NOS1/ILK/EIF2AK3/CDK1/HDAC6/RPS6KA3/CDK5/CDK2/ROCK1/PRKCB/CCNB1/ERN1/APP/AURKB
GO:0034599	cellular response to oxidative stress	TP53/IL6/TNF/MMP9/AKT1/TLR4/JAK2/MMP2/MAPK1/SIRT1/HMOX1/EZH2/JUN/MMP3/MAPK8/NOX4/FOS/IL10/SRC/MPO/PDGFRA/EGFR/STAT6/NOS3/HDAC2/MET/GSTP1/APEX1/G6PD/PARP1/MDM2/GSR/BCL2/MAPK3/RELA/MCL1/BTK/ARG1/ABL1/CYBB/PCNA/NQO1/CDK1/HDAC6/CYP1B1/CDK2/CCNA2/SPHK1
GO:0018209	peptidyl-serine modification	VEGFA/IL6/TNF/AKT1/PTGS2/BRAF/IFNG/MAPK1/MIF/HSP90AA1/MAPK8/PRKDC/TOP1/AKT2/ROCK2/HSP90AB1/TGFBR1/SRC/EGFR/PIK3CA/RAF1/MTOR/MAPK14/RET/SGK1/PDE4D/PARP1/BCL2/MAPK3/PRKACA/IKBKB/RPS6KB1/PLK1/SYK/PRKCA/NOS1/ILK/EIF2AK3/CDK1/HDAC6/RPS6KA3/CDK5/CDK2/ROCK1/PRKCB/CCNB1/ERN1/APP/AURKB
GO:0000302	response to reactive oxygen species	IL6/TNF/MMP9/AKT1/MMP2/MAPK1/SIRT1/HMOX1/EZH2/JUN/MMP3/MAPK8/PDGFRB/FOS/IL10/SRC/MPO/PDGFRA/EGFR/ADA/STAT6/NOS3/HDAC2/MET/GSTP1/CASP3/APEX1/MDM2/BCL2/MAPK3/RELA/BTK/ARG1/ABL1/PCNA/NQO1/CDK1/HDAC6/CYP1B1/CDK2/PTK2B/CCNA2/SPHK1
GO:0070482	response to oxygen levels	TERT/TP53/VEGFA/AKT1/CXCR4/PTGS2/CRHR1/PPARG/CDKN1A/MMP2/SIRT1/HMOX1/EP300/PLAU/VCAM1/PDGFRB/NOX4/ROCK2/KCNK3/SRC/ADA/TEK/SLC6A4/ICAM1/RAF1/NOS2/EDNRA/PLAT/HDAC2/MTOR/CASP3/CYP1A1/PPARA/MYC/ADORA1/MDM2/PSMB5/PSMD3/BCL2/CDK4/MMP14/CYBB/NOS1/CREBBP/NAMPT/SLC2A4/PTK2B/CCNB1/CCNA2/DPP4/ADAM17/HSP90B1/CD38
GO:0033002	muscle cell proliferation	TERT/IL6/TNF/STAT3/MMP9/AKT1/PTGS2/PPARG/IFNG/JAK2/CDKN1A/MMP2/MAPK1/HMOX1/JUN/SHH/IGFBP3/TGM2/PRKDC/PDGFRB/HDAC4/IL10/TGFBR1/ELANE/EGFR/FGFR1/FGFR2/MTOR/MAPK14/GSTP1/COMT/HMGCR/MDM2/RBP4/ILK/NAMPT/CDK1/PTGIR/HTR1B/CCNB1/RXRA/ERN1/S1PR1
GO:0001819	positive regulation of cytokine production	IL6/TNF/STAT3/PTGS2/TLR4/IFNG/JAK2/SERPINE1/IL4/HMOX1/MIF/TLR9/EP300/PTPN11/F2R/PRKDC/F2RL1/IL6ST/ROCK2/MMP12/ADORA2B/IL10/ELANE/SRC/CD40LG/HRAS/PTPN22/PTPRC/GAPDH/STAT6/IL2/HDAC2/MMP8/CASP8/MAPK14/PDE4D/HTR2B/MAPK3/RELA/AGPAT2/P2RX7/CASP1/SYK/ABL1/CYBB/PTGER4/FLT4/EIF2AK3/PDE4B/CREBBP/CYP1B1/CSF1R/PIK3R1/APP/SPHK1/ADAM17
GO:0050727	regulation of inflammatory response	IL6/TNF/MMP9/PTGS2/TLR4/PPARG/JAK2/F2/SERPINE1/IL4/TLR9/MMP3/XIAP/TGM2/PIK3CG/BRD4/IL6ST/ADORA2B/IL10/ELANE/AGTR1/NR1H4/EGFR/ADA/PTPRC/GBA/TEK/NOS2/IL2/MMP8/SELE/MAPK14/GSTP1/ABCC1/PPARA/ADORA1/CMA1/PLA2G2A/NFKBIA/RELA/PLA2G7/ESR1/BTK/NR1H3/PTGER4/FABP4/LYN/RB1/ADORA2A/SPHK1/DNASE1L3
GO:0072593	reactive oxygen species metabolic process	TP53/TNF/STAT3/AKT1/PTGS2/TLR4/IFNG/JAK2/CDKN1A/F2/MMP3/HSP90AA1/PDGFRB/F2RL1/NOX4/HDAC4/ROCK2/HSP90AB1/IL10/AGTR1/MPO/EGFR/ICAM1/NOS3/NOS2/MMP8/MTOR/MAPK14/GSTP1/CYP1A1/INSR/G6PD/TPO/TRPV1/BCL2/P2RX7/SYK/CYBB/NOS1/XDH/NQO1/HDAC6/CYP1A2/CYP1B1/PTK2B
GO:0050673	epithelial cell proliferation	VEGFA/TNF/STAT3/AKT1/ERBB2/PPARG/CCND1/MAPK1/SIRT1/HMOX1/ITGB3/JUN/TP63/SHH/IGFBP3/PRKDC/MMP12/IL10/TGFBR1/AGTR1/KIT/EGFR/HRAS/FGFR1/TEK/STAT6/MAP2K1/SCN5A/F3/FGFR2/MTOR/KDR/CCR3/VDR/MYC/HTR2B/FLT1/ESR1/MMP14/PGR/ARG1/PRKCA/FLT4/RPS6KA1/XDH/RB1/CDK6/PIK3CB/AR/ERN1/GLUL/ADAM17/FAP
GO:0006874	cellular calcium ion homeostasis	CXCR4/CRHR1/JAK2/F2/F2R/TGM2/ITGAV/F2RL1/PIK3CG/CXCR2/KCNK3/ELANE/AGTR1/PDGFRA/CXCR1/PTPRC/EDNRA/IL2/CCR5/CCR3/VDR/PDE4D/ADORA1/HTR2B/TRPV1/BCL2/PRKACA/P2RX7/F2RL3/ESR1/HTR2A/ABL1/PTGER4/NOS1/LYN/CCR1/GRIN2B/CYSLTR1/PLA2G1B/PTGIR/GRIN2A/CDK5/PTK2B/HTR1B/PRKCB/MCHR1/PIK3CB/GPR35/EDN3/APP/CCKBR/HSP90B1/S1PR1/CD38
GO:0034614	cellular response to reactive oxygen species	IL6/TNF/MMP9/AKT1/MMP2/MAPK1/SIRT1/EZH2/JUN/MMP3/MAPK8/FOS/IL10/SRC/MPO/PDGFRA/EGFR/STAT6/NOS3/HDAC2/MET/APEX1/MDM2/MAPK3/RELA/BTK/ARG1/ABL1/PCNA/NQO1/CDK1/HDAC6/CYP1B1/CDK2/CCNA2/SPHK1
GO:0051222	positive regulation of protein transport	TP53/IL6/TNF/ERBB2/PTGS2/TLR4/IFNG/JAK2/MAPK1/MIF/TP63/MAPK8/SHH/F2R/F2RL1/AKT2/MMP12/CFTR/IL10/SRC/NR1H4/EGFR/HRAS/PTPN22/GAPDH/IL2/MMP8/CASP8/MAPK14/GZMB/EZR/MDM2/HTR2B/RBP4/BCL2/MAPK3/PRKACA/P2RX7/CASP1/CHRM1/SYK/ABL1/PTGER4/PLA2G1B/ACHE/CSF1R/CDK5/ADORA2A/PIK3R1/SREBF2/BLK/GLUL/CD38
GO:0007568	aging	TERT/TP53/STAT3/AKT1/PTGS2/CDKN1A/MAPK1/SERPINE1/SIRT1/MIF/JUN/TP63/MMP7/VCAM1/PRKDC/PDGFRB/NOX4/FOS/IL10/MPO/HRAS/ADA/ICAM1/MAP2K1/MTOR/NPM1/MAPK14/APEX1/CYP1A1/PDE4D/HMGCR/BCL2/MAPK3/RELA/MME/ARG1/HTR2A/ABL1/ILK/CASP9/NQO1/NAMPT/CDK1/CDK6/APP/AURKB
GO:0055074	calcium ion homeostasis	CXCR4/CRHR1/JAK2/F2/F2R/TGM2/ITGAV/F2RL1/PIK3CG/CXCR2/KCNK3/ELANE/AGTR1/PDGFRA/CXCR1/PTPRC/EDNRA/IL2/CCR5/CCR3/VDR/PDE4D/ADORA1/HTR2B/TRPV1/BCL2/PRKACA/P2RX7/F2RL3/ESR1/HTR2A/ABL1/PTGER4/NOS1/LYN/CCR1/GRIN2B/CYSLTR1/PLA2G1B/PTGIR/GRIN2A/CDK5/PTK2B/HTR1B/PRKCB/MCHR1/PIK3CB/GPR35/EDN3/APP/CCKBR/HSP90B1/S1PR1/CD38
GO:1904951	positive regulation of establishment of protein localization	TP53/IL6/TNF/ERBB2/PTGS2/TLR4/IFNG/JAK2/MAPK1/MIF/TP63/MAPK8/SHH/F2R/F2RL1/AKT2/MMP12/CFTR/IL10/SRC/NR1H4/EGFR/HRAS/PTPN22/GAPDH/IL2/MMP8/CASP8/MAPK14/GZMB/EZR/MDM2/HTR2B/RBP4/BCL2/MAPK3/PRKACA/P2RX7/CASP1/CHRM1/SYK/ABL1/PTGER4/PLA2G1B/ACHE/CSF1R/CDK5/ADORA2A/PIK3R1/SREBF2/BLK/GLUL/CD38
GO:0070371	ERK1 and ERK2 cascade	TNF/ERBB2/TLR4/BRAF/MAPK1/MIF/JUN/PTPN11/F2R/ITGAV/PDGFRB/F2RL1/NOX4/SRC/PDGFRA/KIT/EGFR/HRAS/PTPN22/PTPRC/TEK/ICAM1/MAP2K1/FGFR2/KDR/GSTP1/EZR/MYC/HMGCR/PLA2G2A/HTR2B/FLT1/MAPK3/SYK/HTR2A/PRKCA/ABL1/PTGER4/LYN/FLT4/CCR1/FLT3/CSF1R/PTK2B/APP
GO:0036293	response to decreased oxygen levels	TERT/TP53/VEGFA/AKT1/CXCR4/PTGS2/CRHR1/MMP2/SIRT1/HMOX1/EP300/PLAU/VCAM1/NOX4/ROCK2/KCNK3/SRC/ADA/TEK/SLC6A4/ICAM1/RAF1/NOS2/EDNRA/PLAT/MTOR/CASP3/CYP1A1/PPARA/MYC/ADORA1/MDM2/PSMB5/PSMD3/BCL2/MMP14/CYBB/NOS1/CREBBP/NAMPT/SLC2A4/PTK2B/CCNB1/CCNA2/DPP4/ADAM17/HSP90B1/CD38
GO:0051480	regulation of cytosolic calcium ion concentration	CXCR4/CRHR1/JAK2/F2/F2R/TGM2/ITGAV/F2RL1/PIK3CG/CXCR2/KCNK3/AGTR1/PDGFRA/CXCR1/PTPRC/EDNRA/IL2/CCR5/CCR3/PDE4D/ADORA1/HTR2B/TRPV1/BCL2/PRKACA/P2RX7/F2RL3/ESR1/HTR2A/ABL1/PTGER4/NOS1/LYN/CCR1/GRIN2B/CYSLTR1/PLA2G1B/PTGIR/GRIN2A/CDK5/PTK2B/HTR1B/MCHR1/GPR35/CCKBR/S1PR1/CD38

**Table 3:** Kyoto Encyclopedia of Genes and Genomes-related genes.

ID	Description	pvalue	Count
hsa05215	Prostate cancer	5.39E−34	42
hsa05161	Hepatitis B	8.55E−30	48
hsa04151	PI3K-Akt signaling pathway	1.73E−29	68
hsa05163	Human cytomegalovirus infection	1.43E−25	51
hsa05205	Proteoglycans in cancer	9.50E−25	48
hsa01522	Endocrine resistance	8.01E−24	34
hsa01521	EGFR tyrosine kinase inhibitor resistance	1.07E−23	31
hsa05167	Kaposi sarcoma-associated herpesvirus infection	1.50E−23	45
hsa04933	AGE-RAGE signaling pathway in diabetic complications	1.72E−23	34
hsa04066	HIF-1 signaling pathway	4.26E−22	34
hsa05212	Pancreatic cancer	8.08E−22	29
hsa05418	Fluid shear stress and atherosclerosis	2.72E−21	37
hsa05223	Non-small cell lung cancer	6.30E−21	27
hsa04210	Apoptosis	1.20E−20	36
hsa05220	Chronic myeloid leukemia	1.30E−20	28
hsa05219	Bladder cancer	2.18E−19	21
hsa05222	Small cell lung cancer	3.94E−19	29
hsa05169	Epstein-Barr virus infection	9.28E−19	41
hsa05235	PD-L1 expression and PD-1 checkpoint pathway in cancer	1.76E−18	28
hsa04068	FoxO signaling pathway	2.79E−18	33
hsa04015	Rap1 signaling pathway	4.13E−18	41
hsa05160	Hepatitis C	1.36E−17	35
hsa05206	MicroRNAs in cancer	1.78E−17	49
hsa05135	Yersinia infection	1.96E−17	32
hsa05214	Glioma	2.66E−17	25
hsa05230	Central carbon metabolism in cancer	5.59E−17	24
hsa05203	Viral carcinogenesis	6.19E−17	39
hsa05210	Colorectal cancer	9.32E−17	26
hsa05218	Melanoma	1.18E−16	24
hsa05166	Human T-cell leukemia virus 1 infection	1.27E−16	40

### 3.6. Construction of the drug–targets–disease network

**Figure 7.**
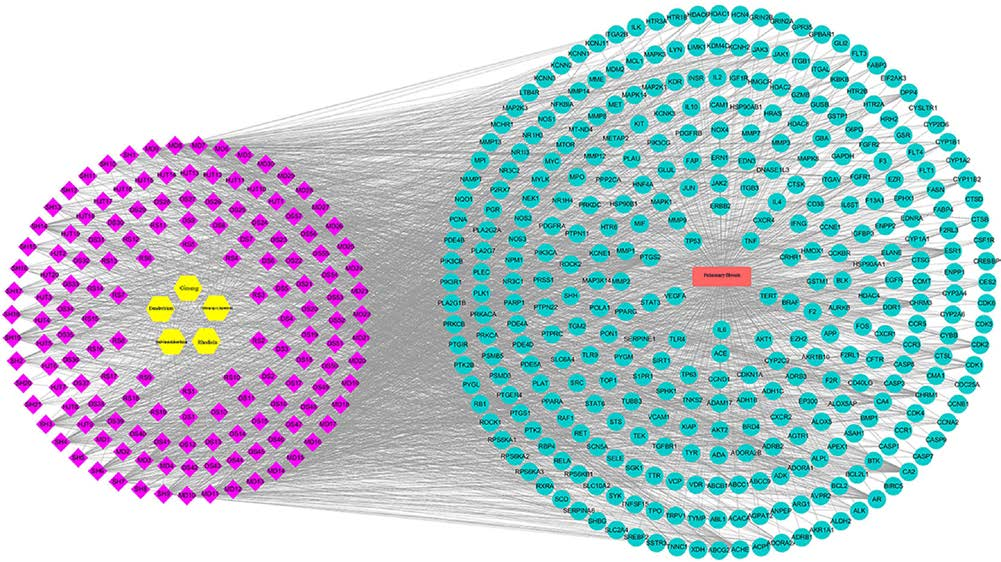
Chinese herbal medicine - active ingredient - disease target network construction.

Import herbs, active ingredients, diseases, and drug–disease common targets into Cytoscape 3.6.1 to obtain the drug–target–disease network (Fig. 7). The active ingredients were ranked by degree value, and those with values greater than the average were selected as key active ingredients, resulting in 45 key ingredients. The network diagram showed that the species with the largest degree value was Dendrobium, followed by Dendrobium and then Rhodiola. The Betweenness Centrality value represents the number of shortest paths between any two nodes, and a larger path number value indicates a more important ingredient (Table 4). A total of 45 key ingredients were obtained, among which the top rankings by degree value were: 5-hydroxy-7,8-dimethoxy-6-methyl-3-(3’,4’-dihydroxybenzyl)chroman-4-one (MD7), bophiopogonanone b (MD3), methyl ophiopogonanone a (MD8), nobiline (SH14), kaempfer (HJT1), etc. Higher degree values represent more important active ingredients, and 6 of the top 10 key ingredients belonged to Ophiopogon japonicus and 3 to Dendrobium, while only 1 belonged to Rhodiola, suggesting that Dendrobium and Dendrobium play a key role in the treatment of pulmonary fibrosis disease.

**Table 4:** Ranking of key components by degree.

Number	Name	Average Shortest Path Length	Betweenness Centrality	Closeness Centrality	Degree
1	MD7	2.589899	0.00859	0.386115	57
2	MD3	2.545455	0.009662	0.392857	56
3	MD8	2.658586	0.005725	0.37614	56
4	SH14	2.618182	0.01076	0.381944	56
5	HJT1	2.561616	0.011808	0.390379	55
6	MD11	2.662626	0.005492	0.375569	54
7	MD20	2.618182	0.00824	0.381944	52
8	SH3	2.577778	0.009196	0.387931	52
9	SH19	2.614141	0.00953	0.382535	51
10	MD18	2.658586	0.00686	0.37614	50
11	MD19	2.614141	0.007506	0.382535	50
12	MD21	2.614141	0.008567	0.382535	50
13	SH6	2.844444	0.005262	0.351563	50
14	SH9	2.557576	0.011141	0.390995	49
15	DS6	2.59798	0.012257	0.384914	48
16	SH4	2.634343	0.007682	0.379601	48
17	SH7	2.581818	0.010203	0.387324	48
18	HJT9	2.808081	0.006286	0.356115	46
19	MD10	2.59798	0.005223	0.384914	45
20	MD9	2.59798	0.005223	0.384914	45
21	MD6	2.610101	0.00505	0.383127	44
22	MD17	2.650505	0.003669	0.377287	41
23	MD13	2.642424	0.003897	0.37844	40
24	MD14	2.59798	0.006511	0.384914	40
25	MD15	2.59798	0.006511	0.384914	40
26	MD22	2.618182	0.006877	0.381944	39
27	SH18	2.662626	0.004407	0.375569	34
28	MD16	2.674747	0.003475	0.373867	33
29	MD12	2.840404	0.001931	0.352063	29
30	DS52	2.735354	0.005101	0.365583	27
31	HJT3	2.711111	0.004322	0.368852	26
32	RS5	2.678788	0.005096	0.373303	26
33	SH1	2.658586	0.002751	0.37614	26
34	SH2	2.658586	0.002751	0.37614	26
35	SH10	2.755556	0.002827	0.362903	25
36	SH17	2.832323	0.002369	0.353067	25
37	HJT2	2.743434	0.002868	0.364507	24
38	HJT13	2.711111	0.002352	0.368852	21
39	MD23	2.844444	9.22E-04	0.351563	20
40	MD24	2.836364	0.001322	0.352564	20
41	SH13	2.836364	0.00136	0.352564	20
42	SH15	2.775758	0.001502	0.360262	20
43	SH5	3.010101	7.43E-04	0.332215	20
44	DS31	2.666667	0.001735	0.375	19
45	MD26	2.99798	5.17E-04	0.333558	18

### 3.7. Pivotal intersection targets and molecular docking

**Figure 8.**
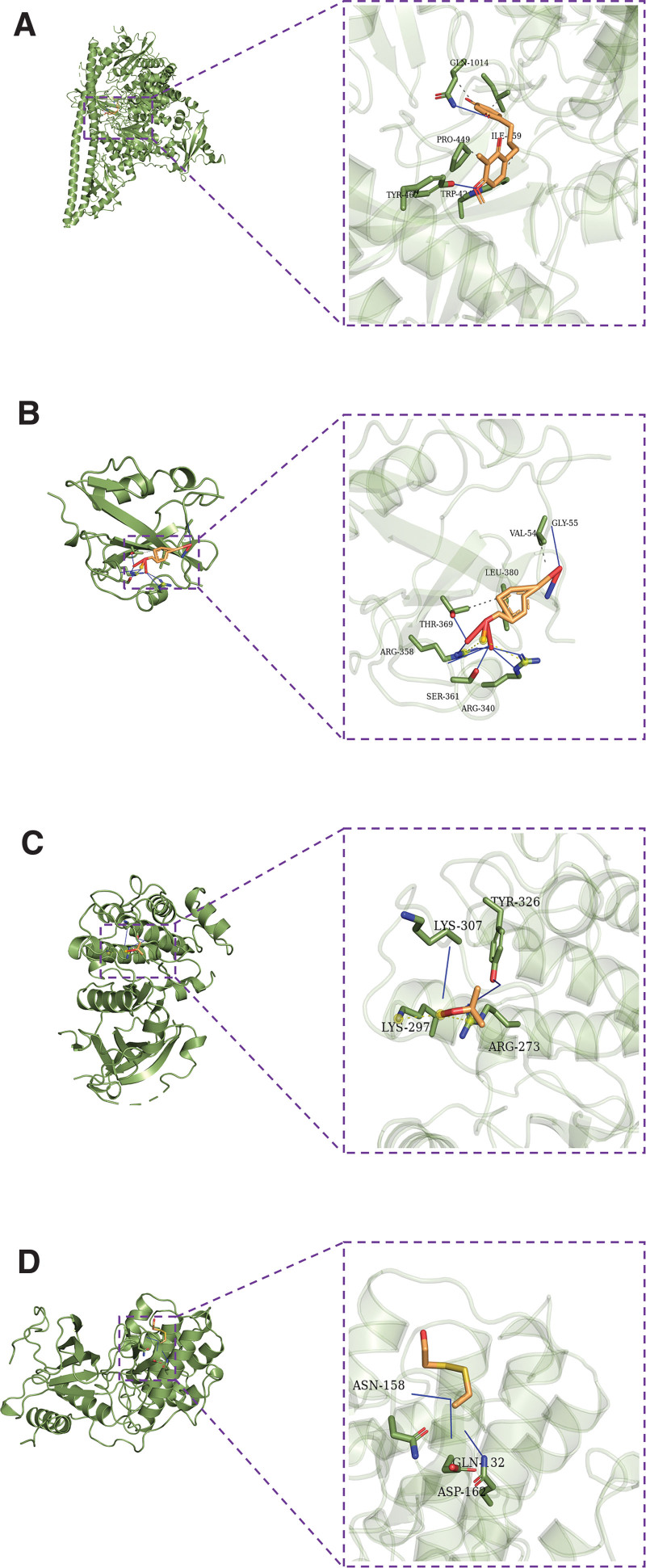
Interaction of (A) PIK3CA, (B) PIK3R1, (C) AKT1, and (D) MAPK1 with 5-hydroxy-7,8-dimethoxy-6-methyl-3-(3′,4′-dihydroxybenzyl) chroman-4-one.

**Figure 9.**
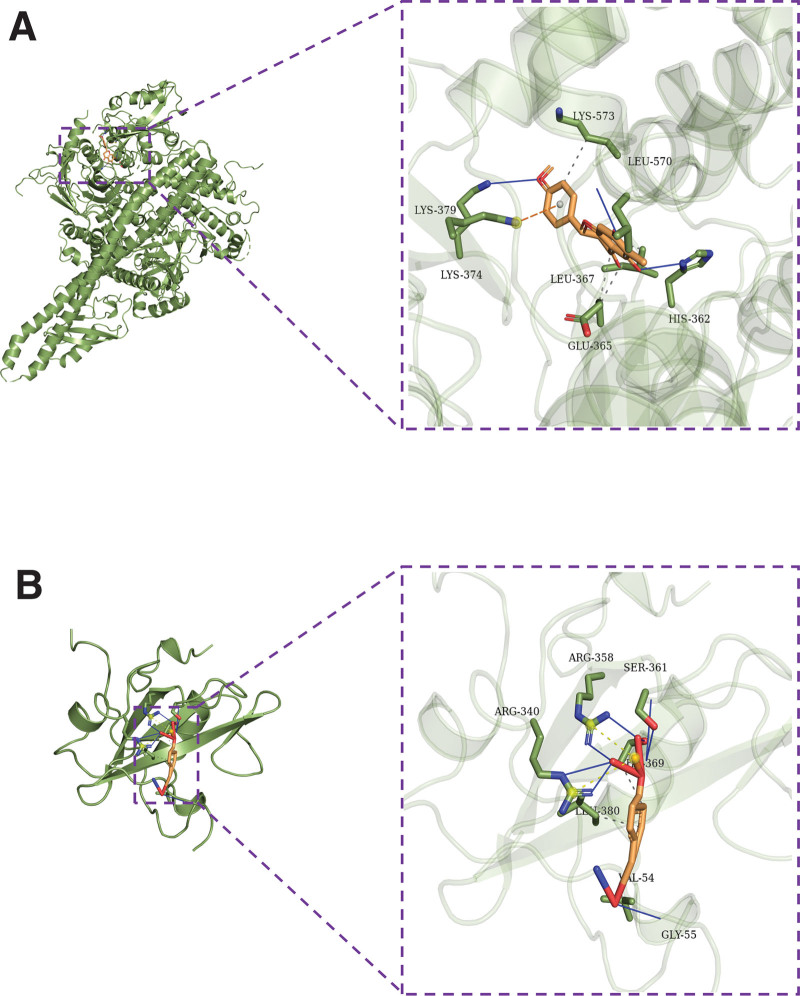
Interaction of (A) PIK3CA and (B) PIK3R1 with bophiopogonanone b.

**Figure 10.**
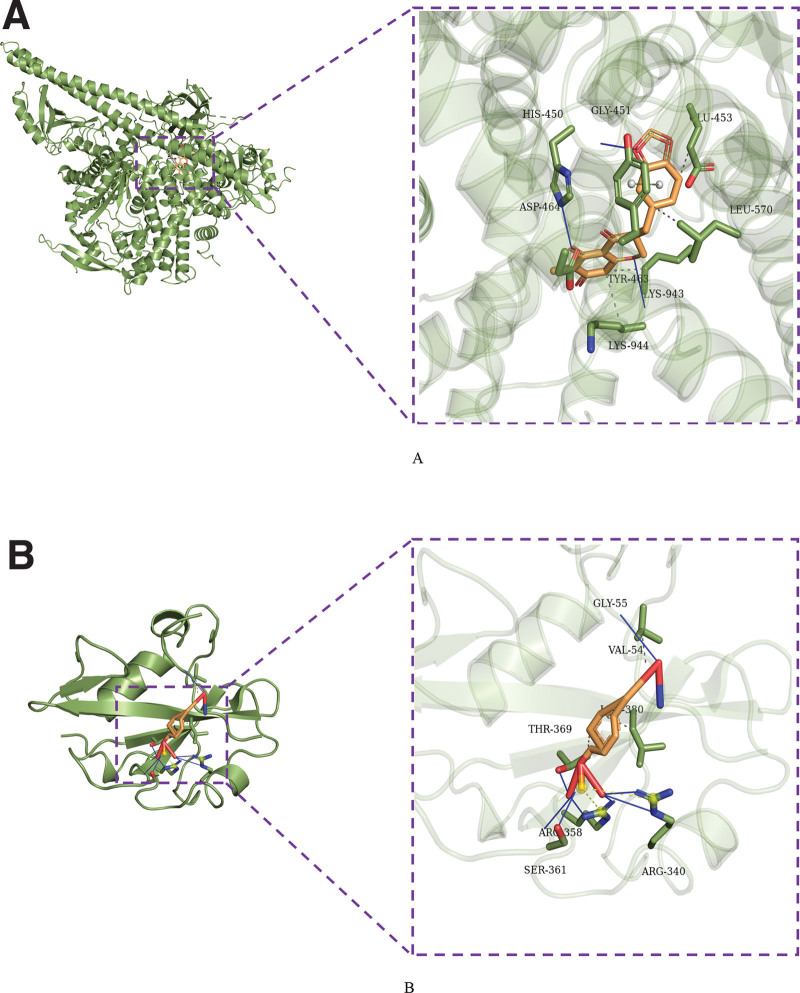
Interaction of (A) PIK3CA and (B) PIK3R1 with methyl ophiopogonanone a.

**Figure 11.**
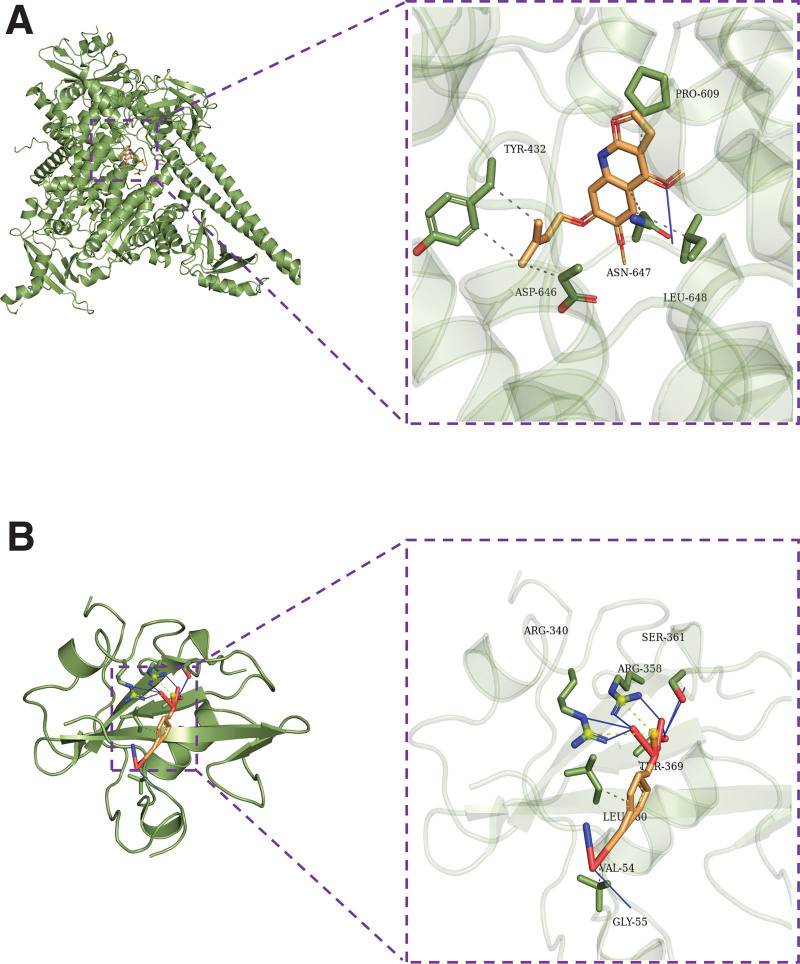
Interaction of (A) PIK3CA and (B) PIK3R1 with nobiline.

We screened the pivotal intersection targets with the Cytohubba tool in Cytoscape software. As shown in Fig. 4, PIK3CA, PIK3R1, MAPK1, SRC, and AKT1 were identified as hub genes in this network and may play a critical role in the therapeutic effect of the Compound Hongginshen Decoction on PF. Molecular docking of these 5 proteins (PIK3CA, PIK3R1, MAPK1, SRC, and AKT1) and their predicted 4 interacting ingredients were performed, including 5-hydroxy-7,8-dimethoxy-6-methyl-3-(3’,4’-dihydroxybenzyl)chroman-4-one (Fig. 8), Bophiopogonanone b (Fig. 9), methyl ophiopogonanone a (Fig. 10), and nobiline (Fig. 11). The binding energy and binding activity of these interacting pairs were summarized in Table 5. The interaction between MD7 and PIK3CA, PIK3R1, MAPK1, SRC, AKT1 requires the docking binding free energy (−7.3, −6.7, −6.2, −4.6, −5.2 kcal/Mol), followed by Bophiopogonanone b and PIK3CA, PIK3R1, MAPK1, SRC, AKT1 (−7.9, −7.1, −7.2, −6.4, −9.7 kcal/Mol), and methyl ophiopogonanone a and PIK3CA, PIK3R1, MAPK1, SRC, AKT1 (−9.5, −8.3, −7.7, −6.5, −7.5 kcal/Mol), and nobiline and PIK3CA, PIK3R1, MAPK1, SRC, AKT1 (−7.6, −6.5, −7.3, −5.9, −7.5 kcal/Mol) indicating a good binding activity in the molecular docking.

**Table 5:** Docking score of key components and core targets.

	PIK3CA	PIK3R1	MAPK1	SRC	AKT1
MD7	−7.3	−6.7	−6.2	−4.6	−5.2
Bophiopogonanone b	−7.9	−7.1	−7.2	−6.4	−9.7
methyl ophiopogonanone a	−9.5	−8.3	−7.7	−6.5	−7.5
nobiline	−7.6	−6.5	−7.3	−5.9	−7.5

## 4. Discussion

In this study, there were 343 common targets between the Compound Hongginshen Decoction and PF. Dysregulation of the PI3K/Akt signaling pathway is seen in a variety of human diseases, including cancer, diabetes, cardiovascular diseases, and neurological diseases, while dysregulation of Akt signaling contributes to an increased frequency of human diseases.^[3–6]^ Another study pointed out that the PI3K-Akt signaling pathway is an important pathway that mediates the pro-survival of multiple growth factors.^[7]^ Based on the herbal-disease-target network analysis, the key ingredients were ranked by degree value, and those with a greater than average degree value were selected as the key active ingredients.

We originally formulated the Compound Hongginshen Decoction mainly for the prevention and treatment of altitude hypoxia, which should be clinically effective in the treatment of chronic obstructive pulmonary disease, significantly improving the symptoms of chronic obstructive pulmonary disease. This network pharmacology study also found that the Compound Hongginshen Decoction has anti-pulmonary fibrosis network pharmacology effects. This decoction is based on the Shengmai Yin and is formulated based on the clinical manifestations of modern pulmonary fibrosis diseases, and scholars and researchers continue to show that Rhodiola, Ginseng, Ophiopogon japonicus, Dendrobium, and Salvia miltiorrhiza exhibit antifibrotic effects. Rhodioloside rosea upregulated the expression of VEGF protein and VEGF-mRNA in lung tissue of rats with pulmonary fibrosis model, enhanced the activity of SOD and CAT enzymes, reduced fibroplasia induced by endothelial inflammatory response, regulated the balance of MPP-2, MPP-9, and TIMP-1, and inhibited the pathological changes and process of pulmonary fibrosis induced by bleomycin.^[8]^ In another study, Rhodiola significantly reduced the expression of α-SMA, a specific marker of fibroblasts, in lung tissues of rats with a pulmonary fibrosis, indicating that Rhodiola significantly inhibited the formation of fibroblasts.^[9]^ A study on idiopathic interstitial lung fibrosis found that ginsenoside Rg1 gavage reduced lung coefficients and α-SMA (α-actinin) levels in IPF (idiopathic interstitial lung fibrosis) rats; ginsenoside Rg1 has some anti-pulmonary fibrosis effects, and the mechanism may be related to the regulation of PDGF-A (platelet-derived growth factor-A) expression.^[10]^ Total ginseng stem saponins may ameliorate BLM (bleomycin) induced experimental pulmonary fibrosis in mice by inhibiting the expression of MMP-2 and MMP-9 proteins in MMPs (matrix metalloproteinases).^[11]^ The previous work found that Ophiopogon japonicus exerted some inhibitory effect on MMP-9 secretion in lung tissue; the decrease in TIMP-1 level in the Ophiopogon japonicus group was significant only at day 28 compared with the model group, suggesting that Ophiopogon japonicus played a role in the significant stage of pulmonary fibrosis.^[12]^ Xu and others pointed out that Dendrobium polysaccharides were related to improving antioxidant capacity and reducing the liver’s inflammatory response in rats.^[13]^ Zhang et al screened the active ingredients of Salvia miltiorrhiza for inhibitory effects on pulmonary fibrosis using an in vitro model of pulmonary epithelial mesenchymal transition and concluded that salvianolic acid A and salvianolic acid B have inhibitory effects on TGF-β1-induced epithelial mesenchymal transition and may be effective ingredients for the treatment of pulmonary fibrosis.^[14]^ In addition, another study found that Ophiopogon japonicus and Salvia miltiorrhiza may have a delayed effect on the development of pulmonary fibrosis by activating superoxide dismutase, inhibiting the scavenging of free radicals, improving the balance between oxidation and antioxidation, reducing cell membrane damage in lung tissue, and weakening the inflammatory response.^[15]^

Fibrosis is scarring and tissue sclerosis caused by excessive deposition of extracellular matrix proteins by myofibroblasts in response to chronic inflammation.^[16]^ A variety of noxious stimuli (including toxins, infectious agents, autoimmune responses, and mechanical stress) can induce fibroblast responses.^[17]^ Fibrosis affects all tissues of the body and, if left unchecked, can lead to organ failure and death.^[18]^

After network topology analysis, the key targets were PIK3CA, PIK3R1, MAPK1, SRC, AKT1, etc., which play important roles in cell growth and apoptosis, in order of core degree from highest to lowest. The PIK3CA gene, which encodes the catalytic subunit p110α of class IA PI3K, can be mutated in a variety of cancers, such as head and neck squamous cell carcinoma, breast cancer, lung cancer, hepatocellular carcinoma, and ovarian cancer, among others.^[19]^ Mutations in PIK3CA in cancer have been shown to be strongly associated with poor prognosis and resistance to standard treatments such as monoclonal antibody therapy and chemotherapy in cancer patients.^[20]^ Not only cancer, PIK3CA is also associated with a variety of fibrotic diseases. In a study by Yang et al on myocardial fibrosis in diabetic mice, it was found that upregulation of miR-203 expression or inhibition of PI3K/Akt signaling pathway could inhibit myocardial fibrosis in diabetic mice through downregulation of PIK3CA expression.^[21]^ PIK3R1, which encodes the regulatory subunit P85αof class I PI3K, inhibits the catalytic activity of P110α kinase and also enhances lipid phosphatase activity by directly binding to PTEN.^[22]^ In addition, aberrant expression of PIK3R1 in cancer promotes the proliferation and invasion of cancer cells and reduces their apoptosis.^[23]^ MAPK1 gene encodes mitogen-activated protein kinase, also known as extracellular signal-regulated kinase, a member of the MAPK family, which is involved in a variety of BPs in the body as a key protein kinase in the signaling cascade.^[24]^ Liu et al proposed that the anti-fibrotic molecule LINC00636 could inhibit MAPK1 by promoting the overexpression of miR-450a-2-3p, which ultimately exerts an ameliorative effect on cardiac fibrosis in patients with atrial fibrillation.^[25]^ Therefore, MAPK1 may be a potential therapeutic target for antifibrotic therapy. Src is a non-receptor tyrosine kinase that integrates multiple extracellular signals and is associated with several human fibrotic diseases by promoting STAT3 phosphorylation to drive fibroblast proliferation. yang et al found that Src enhances the binding of c-Cbl to casein kinase 2 interacting protein 1 (CKIP-1) by promoting c-Cbl phosphorylation and ultimately CKIP-1 protein expression was down-regulated.^[26]^ However, it is interesting to note that CKIP-1 plays a nephroprotective role in the development of diabetic nephropathy. In a study on pulmonary fibrosis, Wang et al found that KX-01 attenuated pulmonary fibrosis by inhibiting the p-Src/p-STAT3 signaling pathway in BLM-induced pulmonary fibrosis rats.^[27]^ Akt1, an isoform of protein kinase B, can be involved in a variety of cellular functions such as survival, metabolism, transcription and translation in the human body after being activated by various signals. Nie et al found that macrophage-specific Akt1 deletion attenuated BLM-induced Idiopathic pulmonary fibrosis and ultimately concluded that Akt1 may regulate pulmonary fibrosis through upregulation of macrophage-derived IL-13.^[28]^ In conclusion the roles and mechanisms of PIK3CA, PIK3R1, MAPK1, SRC, AKT1 in human pulmonary fibrosis are being extensively studied.

In this study, the key targets and active ingredients of the Compound Hongginshen Decoction for the treatment of PF were derived, and therefore the doses of two drugs, Ophiopogon japonicus and Dendrobium, should be increased when using this formula for the treatment of PF. From the perspective of Chinese medicine’s channel entry, Ophiopogon japonicus and Dendrobium belong to the stomach channel, and from the perspective of the 5 Phases, the lung is metal, the stomach is earth, and earth produces metal. Only sufficient stomach qi and body fluids can nourish the lungs and allow their qi, blood, and body fluids to run smoothly. This study is helpful to guide our clinical use of the compound Hongjing Ginseng formula in the treatment of pulmonary fibrosis, and it also suggests that drugs in Chinese medicine with the efficacy of boosting qi and nourishing yin and enriching yin to moisten the lung have the effect of inhibiting pulmonary fibrosis.

However, there are still some shortcomings in this study. On the one hand, the data of this study are mainly based on TCMSP, TCMID and GeneCards, however, many databases exist, and the reproducibility and reliability of these databases are still open to question. On the other hand, although these key genes were initially validated by network pharmacology and molecular docking, further validation by in vivo and in vitro experiments is still needed.

The combination of drugs that invigorate the blood and dissolve stasis and drugs that boost qi and nourish yin may have good efficacy in treating fibrosis. This study established a theoretical basis for the mechanism of action of the Compound Hongginshen Decoction in the clinical application of PF.

## 5. Conclusion

Compound Hongginshen decoction plays a therapeutic role in affecting cell metabolism, anti-inflammation, immune regulation, promoting angiogenesis, and improving anaerobic metabolism. It has multipathway and multitarget effects on the treatment of pulmonary fibrosis. The work further explored its mechanism of PF treatment through TCM network pharmacology and a molecular docking tool. The results suggested that Hongginshen decoction is reasonable and effective, which provides a direction for clinicians to treat PF and a new idea for further research on the mechanism of Hongginshen decoction.

## Author contributions

SWL and LLZ contributed to the conception, design, and commentary of the manuscript. HXC was responsible for manuscript writing. LY was responsible for the collection, assembly, and analysis of the data.

**Data curation:** Yu Lin.

**Software:** Shiwei Liu.

**Writing—original draft:** Haixu Chen.

**Writing—review and editing:** Lianlin Zeng, Shiwei Liu.
